# Simultaneous Combined Endonasal and Transorbital Endoscopic Approach for a Large Orbital Cavernous Venous Malformation: A Technical Case Report

**DOI:** 10.7759/cureus.92109

**Published:** 2025-09-11

**Authors:** Yu Kageyama, Yoshiyuki Kitaguchi, Suetaka Nishiike, Yohei Bamba, Shinichiro Sato

**Affiliations:** 1 Department of Neurosurgery, Osaka Rosai Hospital, Sakai, JPN; 2 Department of Ophthalmology, Osaka University Graduate School of Medicine, Suita, JPN; 3 Department of Otorhinolaryngology - Head and Neck Surgery, Osaka Rosai Hospital, Sakai, JPN

**Keywords:** combined approach, orbital cavernous venous malformation, surgical ergonomics, trans-nasal endoscopic surgery, transnasal endoscopic surgery, transorbital surgery

## Abstract

Orbital cavernous venous malformation (OCVM) is among the most common benign intraconal orbital lesions. Traditionally, ophthalmologists have treated these lesions through a transorbital approach (TOA), while neurosurgeons have favored transcranial access. More recently, the endoscopic endonasal approach (EEA) has become preferred for lesions located inferomedial to the optic nerve. In selected complex cases of orbital lesions, combined approaches integrating EEA and TOA have been employed to overcome the limitations of each method. We report the case of a woman in her 50s with a prior history of transcranial surgery, who presented with complete vision loss in the right eye and exophthalmos due to a large intraconal OCVM. Through collaboration among neurosurgeons, an otorhinolaryngologist, and an oculoplastic surgeon, a simultaneous combined endoscopic approach was undertaken using the EEA and an endoscope-assisted lateral orbitotomy as TOA. Endoscopes were employed in both surgical corridors, enabling circumferential, 360-degree dissection and en bloc removal. Postoperatively, the patient’s right-sided exophthalmos resolved, whereas vision loss remained irreversible. Ptosis, extraocular movement disturbances, and mydriasis developed and gradually improved over time. This case highlights the feasibility of a simultaneous endoscopic EEA and TOA for managing complex intraorbital lesions. Successful execution of such combined approaches requires thorough, meticulous preoperative planning and close multidisciplinary collaboration tailored to each patient.

## Introduction

Orbital cavernous venous malformations (OCVMs) account for approximately 6% of all orbital lesions and are the most common benign orbital tumors in adults [1]. They occur in about 60% of women, suggesting a possible hormonal influence on their clinical course [2], and are histologically characterized as low-flow vascular malformations rather than true neoplasms. Exophthalmos is the most common presenting symptom, followed by diplopia, decreased visual acuity, and ocular motility disturbances. While these lesions are often detected at a small size and can usually be removed en bloc through a single approach, the surgical strategy depends on tumor location. Transcranial approaches have historically been favored by neurosurgeons [3], with the endoscopic endonasal approach (EEA) now offering a less invasive option for inferomedial lesions [4-6], whereas transorbital approaches (TOAs), such as lateral orbitotomy, remain standard for lateral lesions [7-9]. We report a case of a large OCVM previously subjected to craniotomy, which yielded only a biopsy and was followed by progressive enlargement. Because of the presumed adhesions from prior surgery and the lesion’s considerable size, en bloc resection through a single surgical corridor was considered challenging. Therefore, a simultaneous, endoscope-assisted EEA and TOA was performed by a multidisciplinary team comprising neurosurgeons, an otorhinolaryngologist, and an oculoplastic surgeon.

## Technical report

A woman in her 50s who had undergone a craniotomy for resection of an intraorbital tumor at another institution 20 years earlier presented with slowly progressive right-sided exophthalmos and decreased visual acuity. The previous surgery had achieved only a biopsy, and no definitive diagnosis was established at that time. At the time of presentation, the patient had complete vision loss in the right eye. Clinical examination revealed right exophthalmos, lateral globe displacement, and restricted adduction. The right pupil measured 3 mm and was non-reactive to light, while the left pupil was 2 mm with a normal light reflex. Fundoscopy demonstrated optic nerve atrophy in the right eye.

**Figure 1:**
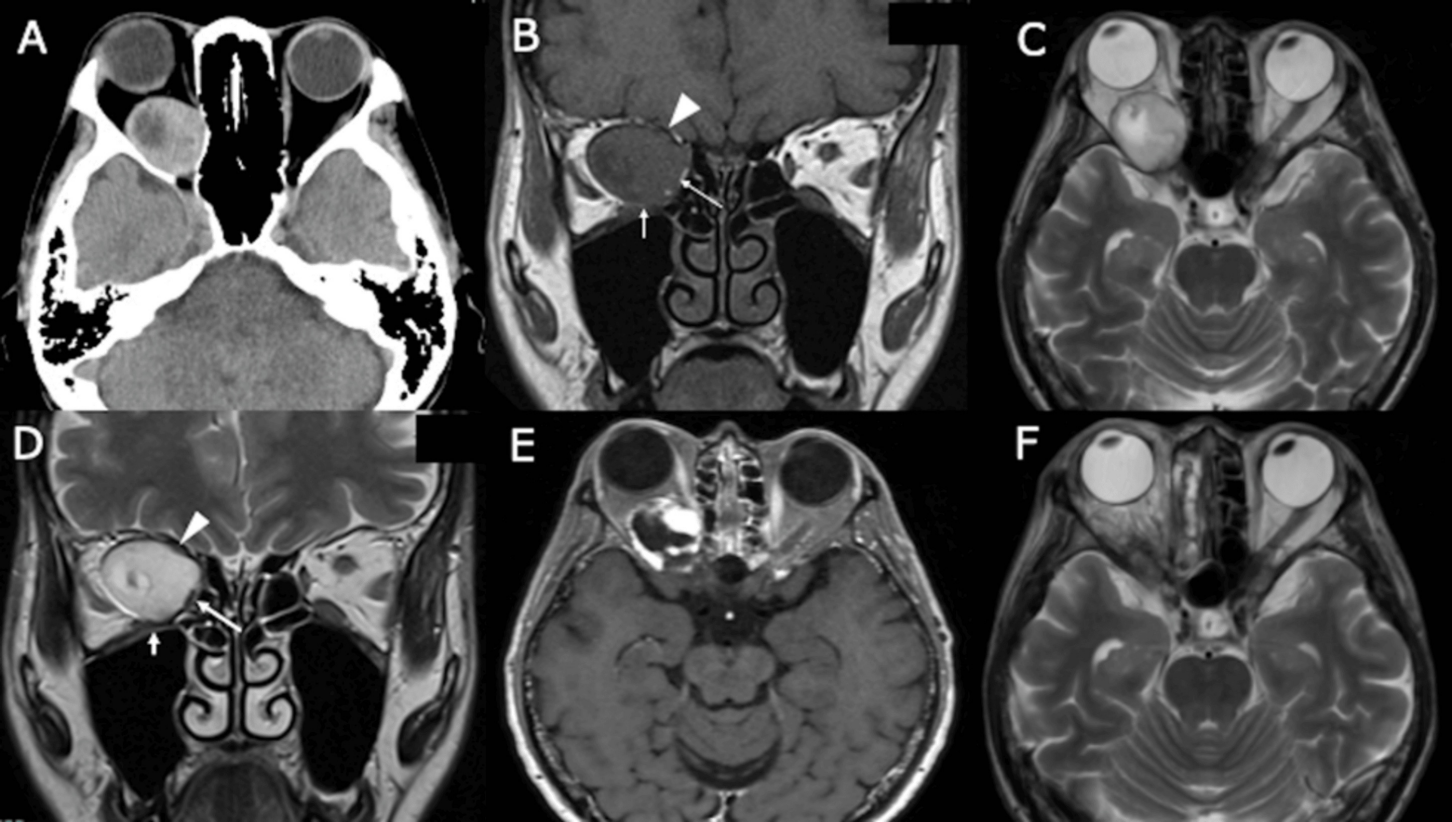
Radiological images (A) Preoperative axial cranial CT scan showing an isodense retrobulbar mass in the right orbit, causing anterior displacement of the globe. (B-E) Preoperative MRI demonstrating an oval-shaped intraorbital lesion with isointensity on T1-weighted imaging (B) and hyperintensity on T2-weighted imaging (C, D). Coronal views (B, D) show compression and thinning of the medial rectus muscle (long arrow) and inferior rectus muscle (short arrow), with a narrow intermuscular corridor between them. The lesion displays heterogeneous delayed contrast enhancement in the late phase (E). The optic nerve (arrowhead) is compressed superiorly and appears thinned. (F) Postoperative MRI confirming total resection of the lesion and resolution of exophthalmos.

CT revealed a well-defined, oval-shaped, isodense retrobulbar mass with a central hypodense area in the right orbit, causing anterior displacement of the eyeball (Figure 1A). MRI demonstrated a 30 × 26 × 21 mm intraconal lesion that was isointense on T1-weighted images and hyperintense on T2-weighted images, with a mixed-intensity core and heterogeneous delayed contrast enhancement (Figure 1B-1E). The optic nerve was compressed superiorly, and the medial rectus muscle was displaced medially and appeared thinned (Figure 1B, 1D). Radiological features were consistent with an intraconal OCVM.

Preoperative assessment suggested that the lesion, particularly along its superior aspect, was adherent to surrounding tissues as a result of the prior craniotomy and biopsy. In addition, the surgical corridor between the medial and inferior rectus muscles appeared narrow, making complete resection through an EEA alone highly challenging. Therefore, a combined strategy incorporating a lateral TOA was planned. The EEA was designed to provide access for dissection from the medial side of the lesion, extending to its superior, inferior, and posterior aspects, while the TOA via lateral orbitotomy was intended to permit dissection from the lateral side toward the superior, inferior, and anterior aspects. By complementing each other’s limitations, these two approaches together enabled true circumferential dissection, thereby facilitating safe and effective en bloc removal. The operating room was arranged to allow simultaneous use of both approaches under endoscopic visualization.

Surgical procedures and clinical course

**Figure 2:**
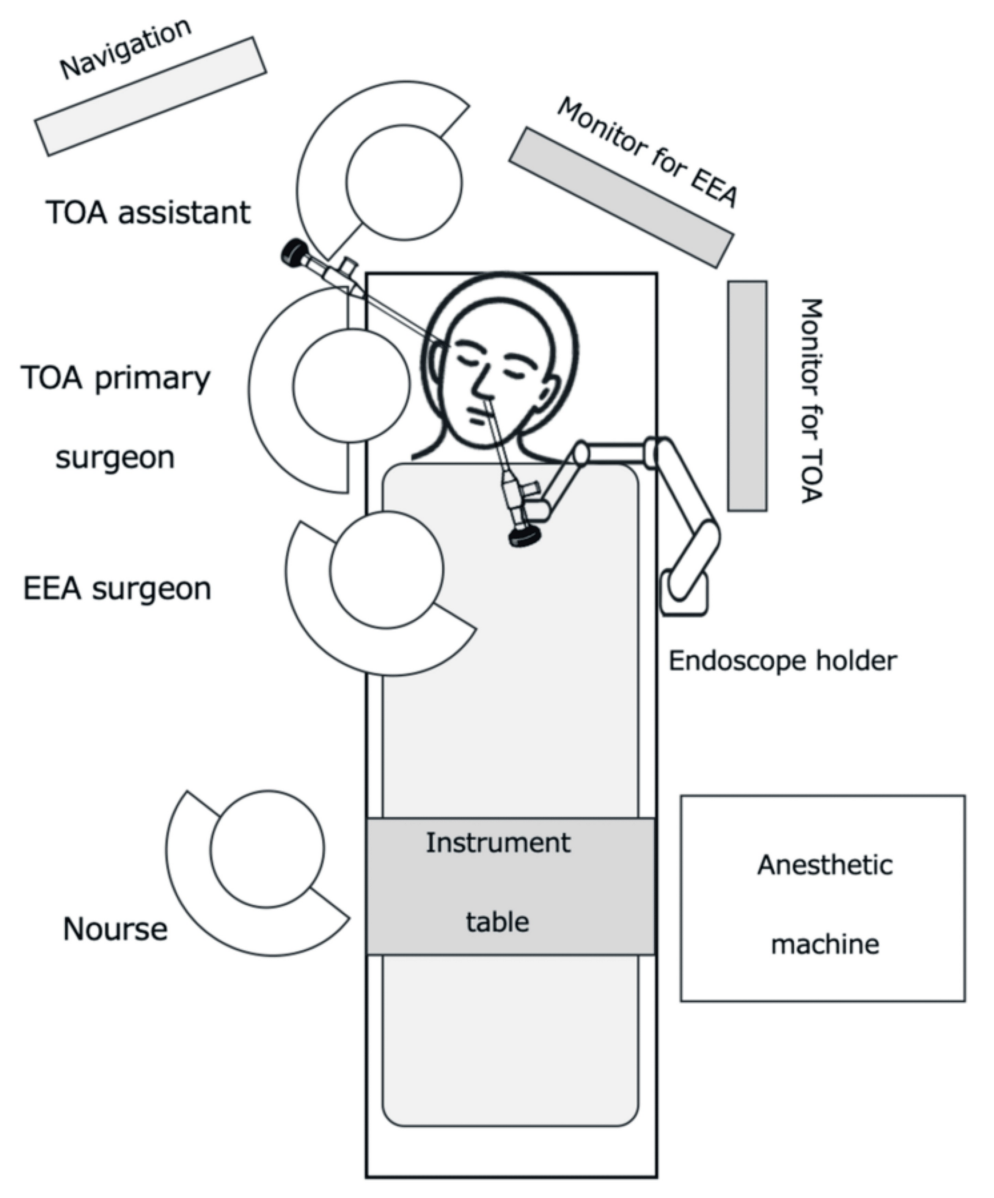
Operating room setup for simultaneous combined endonasal and transorbital endoscopic approach Endoscopic monitors are positioned directly in front of both the endonasal and transorbital surgeons to optimize visualization. EEA, endoscopic endonasal approach; TOA, transorbital approach Image credit: Yu Kageyama

Under general anesthesia, the patient was positioned supine with the head slightly tilted to the left and supported by a donut-shaped cushion. An electromagnetic navigation system was set up. Figure 2 depicts the layout of the surgical teams and equipment in the operating room.

**Figure 3:**
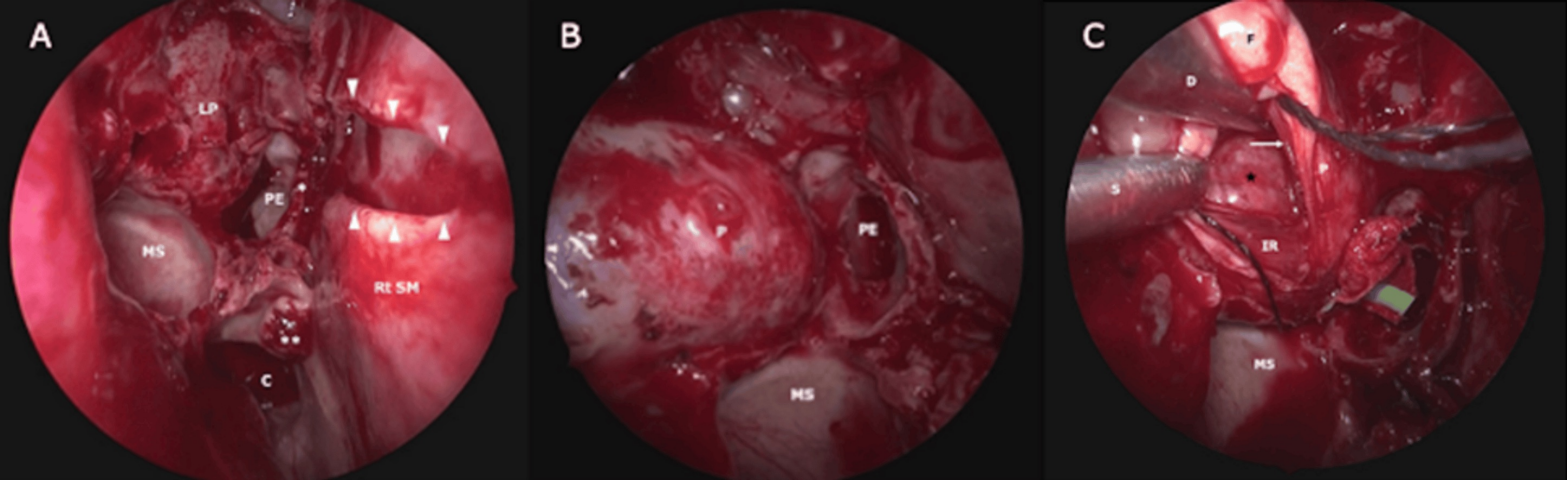
Endoscopic images of the binostril endonasal approach (A) Endoscopic overview of the surgical field in the right nasal cavity after sphenoethmoidectomy and middle/superior turbinectomies. (B) Magnified view of the medial orbital wall. The right lamina papyracea was removed, exposing the periorbita. (C) Dissection of the lesion through the corridor between the medial and inferior rectus muscles. The medial rectus muscle appears thinned due to compression by the CVM. Asterisk = the resected stump of the superior turbinate; double asterisk = the resected stump of the middle turbinate; arrowheads = the horizontal cut of the right septal mucosa; star = the inferomedial aspect of the CVM; arrow = medial rectus muscle C, choana; CVM, cavernous venous malformation; D, dissector; F, intraorbital fat tissue; IR, inferior rectus muscle; LP, lamina papyracea; MS, maxillary sinus; P, periorbita; PE, posterior ethmoid sinus; S, suction tube

The procedure began with an EEA performed by a neurosurgeon and an otorhinolaryngologist. A right sphenoethmoidectomy was carried out to expose the right lamina papyracea. A Killian incision was made on the left septal mucosa, followed by subperiosteal dissection to remove the bony nasal septum. To facilitate the binostril approach, a horizontal septal mucosal incision and middle and superior turbinectomy were performed on the right side. The right lamina papyracea was resected (Figure 3A), and the periorbita was incised horizontally between the medial and inferior rectus muscles. 

Although the intermuscular corridor was narrow, partial dissection of the lesion was achieved from the medial aspect toward its superior, inferior, and posterior directions (Figure 3C). However, consistent with the preoperative prediction, circumferential dissection beyond the equatorial plane at the superior aspect could not be accomplished through the EEA alone.

**Figure 4:**
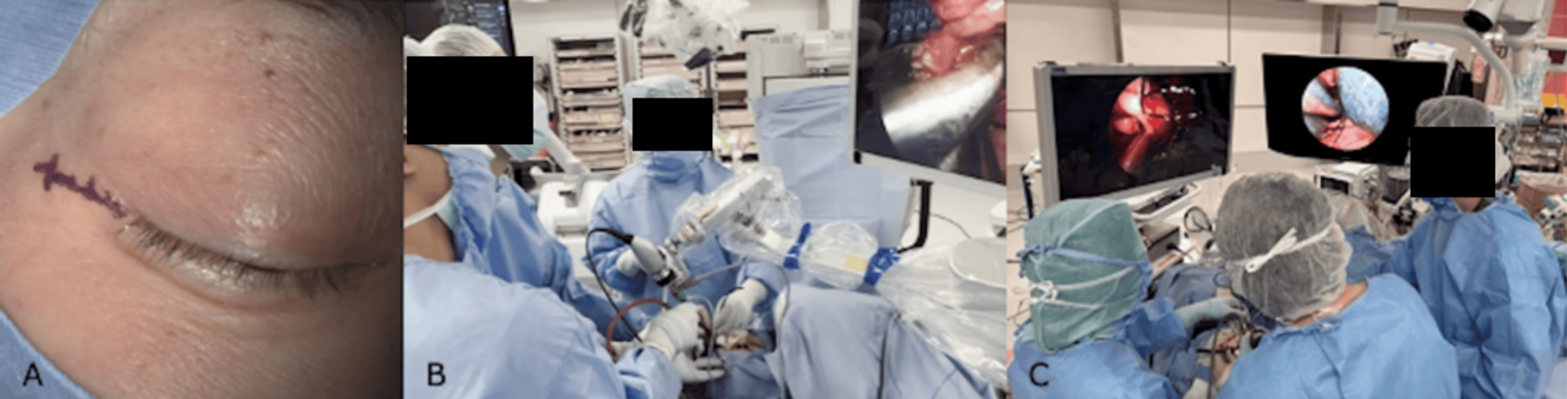
Intraoperative photographs (A) A 1.5 cm lateral canthotomy incision made along a natural skin crease on the right side. (B) Endoscopic TOA performed using a three-surgeon, five-handed technique. (C) Simultaneous combined endonasal and transorbital endoscopic approach. The neurosurgeon performed the EEA using a pneumatic endoscope holder, while the oculoplastic surgeon carried out the TOA with another endoscope in one hand and a surgical instrument in the other. An assistant retracted orbital contents using two malleable ribbon retractors. EEA, endoscopic endonasal approach; TOA, transorbital approach

Next, a lateral orbitotomy was initiated by an oculoplastic surgeon using the Berke technique [7] under a surgical microscope. A 1.5 cm lateral canthotomy incision was made along a natural skin crease (Figure 4A). The lateral canthal tendon was released, and the temporalis muscle was dissected in a subperiosteal plane. The lateral orbital rim and a segment of the lateral orbital wall were removed en bloc using a bone saw, with no additional bony removal required. The periorbita was incised parallel to the lateral rectus muscle, and the dissection was started using a four- to five-handed technique under endoscopic visualization provided by an endoscope mounted on a pneumatic holder (UniArm^®^, Mitaka Kohki Co., Tokyo, Japan). The primary surgeon performed bimanual dissection, while an assistant retracted orbital contents using two malleable ribbon retractors, and a second assistant managed suction and endoscope positioning (Figure 4B).

**Figure 5:**
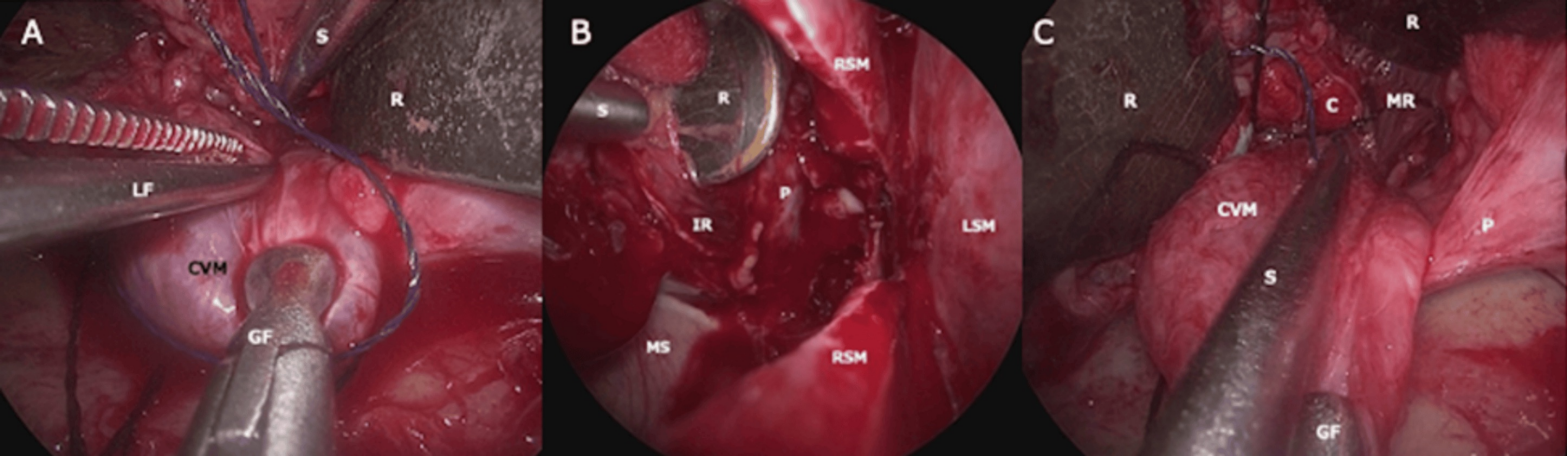
Endoscopic images during the dissection of the lesion (A) Endoscopic view through the TOA via lateral orbitotomy. Three surgeons participated in the dissection: the oculoplastic surgeon manipulates the CVM using GF and LF; one assistant retracts orbital contents using malleable ribbon retractors (R); and another maintains the clear surgical field using a suction tube (S). (B) Endoscopic view from the left nostril, crossing the RSM after horizontal incision. The completed dissection between the CVM and the IR is shown. A malleable ribbon retractor (R), inserted via the transorbital route, elevates the muscle. (C) En bloc resection of the CVM via the transorbital route under endoscopic visualization. C, cottonoid; CVM, cavernous venous malformation; GF, grasping forceps; IR, inferior rectus muscle; LF, Lucae forceps; LSM, left septal mucosa; MR, medial rectus muscle; MS, maxillary sinus; P, periorbita; R, malleable ribbon retractor; RSM, right septal mucosa; S, suction tube; TOA, transorbital approach

For the simultaneous EEA and TOA, the neurosurgeon continued the EEA using the mounted endoscope, while the oculoplastic surgeon conducted the TOA using a second endoscope in one hand and a surgical instrument in the other, assisted by an additional assistant (Figure 4C). Through the TOA, the lateral aspect of the lesion was identified using a corridor between the lateral and inferior rectus muscles created with malleable ribbon retractors. As planned preoperatively, dissection was then advanced from the lateral side toward the superior, inferior, and anterior aspects, complementing the medial dissection achieved via the EEA (Figure 5A). 

**Figure 6:**
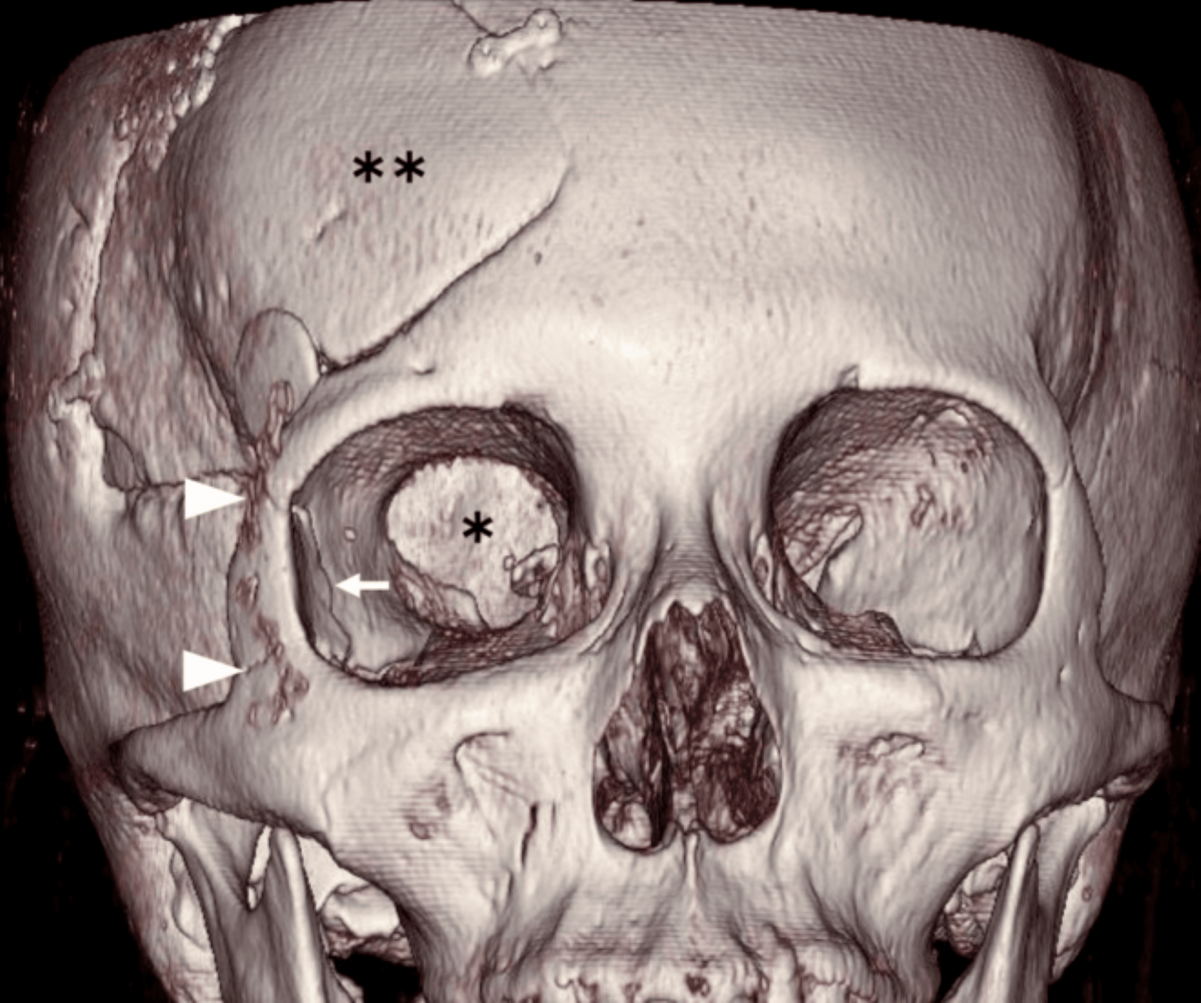
Postoperative three-dimensional CT image of the skull The lateral orbital rim was reconstructed using two resorbable plates without defect of the lateral wall of the right orbit (arrow). Note that after a previous craniotomy, the bone flap (double asterisks), titanium plates, and a ceramic button covering the keyhole were shown, and the superior orbital fissure (asterisk) was enlarged. Arrowhead osteotomy line of the lateral orbital rim in lateral orbitotomy.

As anticipated, adhesions were encountered between the lesion and connective tissues surrounding the optic nerve, which were carefully dissected. With these adhesions released, circumferential dissection was ultimately achieved without exposure of the optic nerve itself (Figure 5B), and the lesion was successfully removed en bloc through the TOA route (Figure 5C). The lateral orbital rim was reconstructed using resorbable plates (Figure 6). NasoPore^®^ (Stryker Japan K.K., Shinjuku, Japan) was placed in the posterior ethmoid sinus to gently compress the exposed periorbita and orbital fat laterally, reducing the risk of postoperative adhesions.

Histopathological examination confirmed a diagnosis of cavernous venous malformation. Postoperatively, the patient’s exophthalmos on the right eye immediately resolved, from 18 mm to 13 mm of the distance from the interzygomatic line to the anterior surface of the globe; however, ptosis, mydriasis, and disturbances of extraocular movement in all directions due to injury to the oculomotor nerve and manipulation of the lateral rectus muscle were observed. Vision loss was irreversible due to preexisting optic atrophy. Six months postoperatively, the ptosis and extraocular movement disturbances had partially improved, and the mydriasis also showed noticeable improvement, with the pupil diameter decreasing from 6.0 mm to 4.5 mm.

## Discussion

In recent years, the EEA has gained popularity for managing orbital lesions, supported by a growing body of literature [4-6]. Classification systems such as the clock-face model [5] and the four-zone model [10] have been introduced to guide surgical approach selection based on a lesion’s location relative to the optic nerve on coronal imaging. Both models consistently suggest that EEA is best suited for lesions situated inferomedial to the optic nerve and extending toward the orbital apex. In contrast, the TOA has traditionally been used by ophthalmologists and orbital surgeons. This approach includes variations such as lateral orbitotomy, transconjunctival access, and superior eyelid incisions [2,8,11]. Among these, lateral orbitotomy is one of the most established techniques, originally described by Krönlein in 1888 [12] and subsequently refined. Berke’s modification, which utilizes a natural lateral canthal crease for the incision, improves cosmetic outcomes [7,13]. A 1.5-cm incision typically avoids injury to the frontal branch of the facial nerve [9].

In this case, the lesion extended to the orbital apex and caused marked inferior displacement of the medial rectus muscle, resulting in a narrowed surgical corridor via the EEA. The patient’s history of prior transcranial surgery also raised concerns regarding adhesions between the lesion and adjacent structures, particularly the optic nerve. Under these conditions, dissection beyond the equatorial plane to the contralateral side would have been difficult and potentially hazardous if attempted through a single surgical corridor. To preserve visual function, an additional surgical route was considered advantageous. Accordingly, a combined approach incorporating both EEA and TOA was employed to achieve safe and effective circumferential dissection. The binostril EEA allowed for precise bimanual dissection, while the lateral orbitotomy provided a wider surgical field and direct lateral access to the lesion. Dissection along the superior aspect of the lesion proved technically challenging with EEA alone, as adhesions around the optic nerve were indeed present. By combining the two approaches, 360-degree circumferential dissection was achieved, permitting en bloc removal. Reconstruction of the lateral orbital wall was unnecessary, as repositioning the orbital rim restored anatomical continuity without a residual defect (Figure 6). Reconstruction of the medial orbital wall remains a subject of debate and may depend on the extent of the defect [14,15]. Postoperatively, the patient developed transient oculomotor nerve palsy and lateral rectus dysfunction. These complications were likely due to the large size of the lesion and the fact that it was a vascular tumor not amenable to debulking, which necessitated a more invasive manipulation than a single surgical approach, particularly in relation to the surrounding critical structures.

Lateral orbitotomy is typically performed under a surgical microscope; however, aligning the optical axis with the dissection plane often requires frequent repositioning of the microscope, and therefore, simultaneous EEA and TOA procedures may involve overlapping fields and limited working space, potentially causing interference between surgical teams. To address these challenges, we employed an endoscope-assisted TOA. By using endoscopes in both corridors, each surgical team was able to operate while viewing separate monitors positioned in front of them (Figure 4C). This technique not only demonstrates the feasibility of 360-degree dissection but also highlights the advantages of dual endoscope collaboration. Although similar combined approaches have been reported for complex skull base lesions primarily located in the intracranial space or paranasal sinuses, no reports have focused on pure intraorbital lesions [10,16-18], nor has lateral orbitotomy been described as a TOA combined with an EEA. Therefore, the present case, utilizing simultaneous endoscopic assistance through both an EEA and lateral orbitotomy for resection of a large OCVM, represents a unique approach.

Surgical approaches to orbital lesions should be individualized based on lesion-specific characteristics, with careful consideration of access, invasiveness, and cosmetic outcomes. While single-approach strategies are generally preferred, complex cases, such as the present one, may benefit from a combined approach. Among the available combinations, pairing an EEA with a lateral orbitotomy offers an optimal balance of surgical exposure, minimal invasiveness, and favorable cosmetic results. Successful implementation of such combined strategies requires meticulous multidisciplinary planning involving ophthalmologists, neurosurgeons, otorhinolaryngologists, and oculoplastic surgeons.

It is important to recognize the limitations and risk-benefit profile of this approach. The procedure requires two endoscopic systems, duplicate surgical instrument sets, and preoperative simulation of the operating room setup; therefore, it may not be feasible in all institutions. Moreover, because the indications for this approach are limited, the learning curve remains relatively shallow. In particular, when the TOA is performed by surgeons other than experienced oculoplastic specialists, prior training, such as cadaveric dissection, is recommended.

## Conclusions

We presented a case of a large OCVM successfully treated using a simultaneous, combined EEA and lateral orbitotomy. Although this approach is more invasive and requires complex preoperative preparation, effective multidisciplinary collaboration enabled 360-degree circumferential dissection and en bloc removal, underscoring its potential utility in selected cases. Future accumulation of cases and longer follow-up will be essential to refine patient selection, assess long-term outcomes, and better define the role of this combined approach in the surgical management of intraorbital lesions.
